# Anatomical basis for contribution of hip joint motion by the obturator internus to defaecation/urinary functions by the levator ani via the obturator fascia

**DOI:** 10.1111/joa.13810

**Published:** 2022-12-18

**Authors:** Satoru Muro, Akimoto Nimura, Takuya Ibara, Kenro Chikazawa, Masataka Nakazawa, Keiichi Akita

**Affiliations:** ^1^ Department of Clinical Anatomy Tokyo Medical and Dental University Tokyo Japan; ^2^ Department of Functional Joint Anatomy, Graduate School of Medical and Dental Sciences Tokyo Medical and Dental University Tokyo Japan; ^3^ Department of Obstetrics and Gynecology, Saitama Medical Center Jichi Medical University Saitama Japan; ^4^ Faculty of Health Sciences Tokyo Ariake University of Medical and Health Sciences Tokyo Japan

**Keywords:** anatomy, defaecation, hip joint muscle, pelvic floor, urination

## Abstract

The functional association between hip joint motion and defaecation/urinary function has attracted considerable research and clinical attention owing to the potential novel approaches for pelvic floor rehabilitation; however, the anatomical basis remains unclear. This study, therefore, aimed to analyse the anatomical basis of force transmission between the obturator internus, a muscle of the hip joint, and the levator ani, a muscle of the pelvic floor. Twenty‐three cadavers were used for macroscopic and histological analyses. The three‐dimensional structures of the muscles and fascia were recorded using a high‐definition camera and a 3D scanner. The arrangement and attachment of the muscle fibres, tendons and fascia were visualised using histological sections stained with Masson's trichrome. The obturator internus and levator ani were in broad contact through the obturator fascia. The height of their contact area was 24.6 ± 9.1 mm. Histologically, the obturator internus and levator ani shared a large area of the obturator fascia, and the obturator fascia provided the attachment of several muscle layers of the levator ani. The contribution of hip joint motion to defaecation/urinary function can be explained by the broad ‘planar’ contact between the obturator internus and levator ani. This anatomical feature suggests that movement of the obturator internus creates the foundation for the function of the levator ani and contributes to pelvic floor support through the obturator fascia. This study provides an anatomical basis for the effectiveness of the hip muscles in improving defaecation/urinary function by enabling balanced and proper movements.

## Graphical abstract

This study clarified a broad ‘planar’ contact between the obturator internus, a muscle of the hip joint, and the levator ani, a muscle of the pelvic floor. This anatomical feature suggests that movement of the obturator internus creates the foundation for the function of the levator ani and contributes to pelvic floor support. This finding provides an anatomical basis for the effectiveness of the hip muscles in improving defaecation/urinary function through enabling balanced and proper movements.

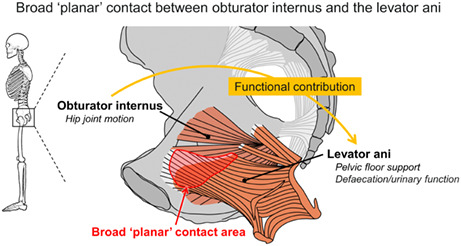

## 1 INTRODUCTION

The rehabilitation of the lower limb and pelvic floor muscles is designed and implemented separately because the anatomy and function of the lower limb and pelvic floor are discussed in separate frames, and there is little knowledge to relate them. Among the muscles of the lower limb, one of the least recognised anatomical structures, also known as the ‘secret’ muscle, is the obturator internus. The obturator internus is one of the six deep lateral rotator muscles of the hip and is responsible for the external rotation and abduction of the hip joint (Neumann, 2010). Most muscle is hidden inside the hip bone; therefore, it is difficult to determine its shape from the surface of the body. Interestingly, although the obturator internus is a lower limb muscle, it is also a component of the pelvic wall.

Several recent reports have shown a functional relationship between the obturator internus and pelvic floor muscles, and the relationship between hip joint motion and defaecation/urinary functions has been discussed. For example, Tuttle et al. (2016) compared a group of women who performed hip external rotation exercises with a control group and reported that the rehabilitation of the obturator internus improved pelvic floor muscle strength for defaecation/urinary functions. Two studies also evaluated patients with urinary incontinence who underwent total hip arthroplasty and reported that hip joint treatment contributed to improved urinary incontinence (Okumura et al., 2017; Tamaki et al., 2014). Such a phenomenon in clinical practise suggests a structural relationship between the hip joint and pelvic floor muscles. Therefore, it is necessary to understand the structural relationship between the hip and pelvic floor muscles to elucidate the mechanism of this functional linkage between hip joint motion and the pelvic floor.

The obturator internus, which is one of the rotator muscles of the hip joint, is composed of the pelvic wall and is in contact with the levator ani, the largest of the pelvic floor muscles (Neumann, 2010; Standring & Gray, 2015). Conventional textbooks, such as ‘Cunningham's textbook of anatomy’, state that the obturator internus and levator ani are in contact via a linear structure called the tendinous arch of levator ani (Cunningham & Romanes, 1981). However, such a limited ‘linear’ contact cannot adequately explain the clinical phenomena described above. Therefore, we hypothesized that the obturator internus and levator ani would have a broader contact area than is previously described. The functional relationship between the obturator internus and levator ani is implied if broad contact is shown. Therefore, this study aimed to clarify the structural relationship between the obturator internus and levator ani, focusing on the close contact area and origin of the levator ani.

## 2 MATERIALS AND METHODS

### 2.1 Preparation of cadaveric specimens

Twenty‐three cadavers (12 males and 11 females; mean age at death, 77.1 years; age range, 42–94 years) were donated to our department. The donation document format was congruent with the Japanese law entitled ‘The Act on Body Donation for Medical and Dental Education’ (Act No. 56 of 1983). Before their death, all donors voluntarily declared that their remains would be donated as materials for education and study. At that time, the purpose and methods of using body donor corpses were explained, and informed consent was obtained. After their death, we explained the informed consent to the bereaved families and confirmed that there was no opposition. All cadavers were fixed via arterial perfusion with 8% formalin and preserved in 30% alcohol. The study was approved by the Board of Ethics at Tokyo Medical and Dental University (approval number: M2018‐006). All methods were performed following the relevant guidelines and regulations.

### 2.2 Macroscopic anatomy

Nineteen cadavers were used for macroscopic examinations. From the 12 cadavers, 14 pelvic halves (2 whole pelvises, 4 right pelvic halves and 6 left pelvic halves) were obtained to dissect the obturator internus and levator ani. These two muscles and the surrounding connective tissues were observed from the medial and posterior aspects, focusing on their placement, the relationship between the two and the associated fascial structures. One of the dissected pelvic halves was captured using a 3D scanner (EinScan‐SE; Japan 3D Printer Co., Ltd., Tokyo, Japan) and observed on a three‐dimensional image. Whole pelvises were sectioned in the oblique coronal plane parallel to the anal canal using the remaining seven cadavers. Since the levator ani pulls the anal canal anterosuperiorly, sectioning in such a plane parallel to the anal canal provides a cross‐section parallel to the muscle bundles at the origin of the levator ani. Sections of the obturator internus and levator ani were examined. We measured (1) the height of the contact area between the obturator internus and levator ani, (2) the maximum thickness of the obturator internus, (3) levator ani, (4) the width of the ischioanal fossa (horizontal distance from the inferior end of the obturator internus to the levator ani) and (5) the depth of the ischioanal fossa (distance from the superior vertex of the ischioanal fossa to the inferior end of the obturator internus) using ImageJ (version 1.52; National Institutes of Health, Bethesda, Maryland, United States of America) (Schneider et al., 2012).

### 2.3 Histology

Four cadavers were used for histological examination. To histologically observe the contact area between the obturator internus and levator ani, tissue blocks were collected in cross‐sections parallel to the muscle fibres of the levator ani using a diamond band pathology saw (EXAKT 312; EXAKT Advanced Technologies). These tissue blocks were fixed in 10% formalin, decalcified with Plank–Rychlo solution (AlCl_3_:6H_2_O 126.7 g/L, HCl 85 ml/L, HCOOH 50 ml/L) and dehydrated. The blocks were embedded in paraffin and sectioned into 5‐μm‐thick specimens. The histological sections were then stained with Masson's trichrome. Finally, the stained specimens were scanned as entire slides using a high‐quality scanner (GT‐X980, EPSON, Japan). Additionally, local high‐magnification digital pictures were acquired using a digital slide scanner (NanoZoomer‐SQ C13140, HAMAMATSU, Japan).

## 3 RESULTS

**FIGURE 1:**
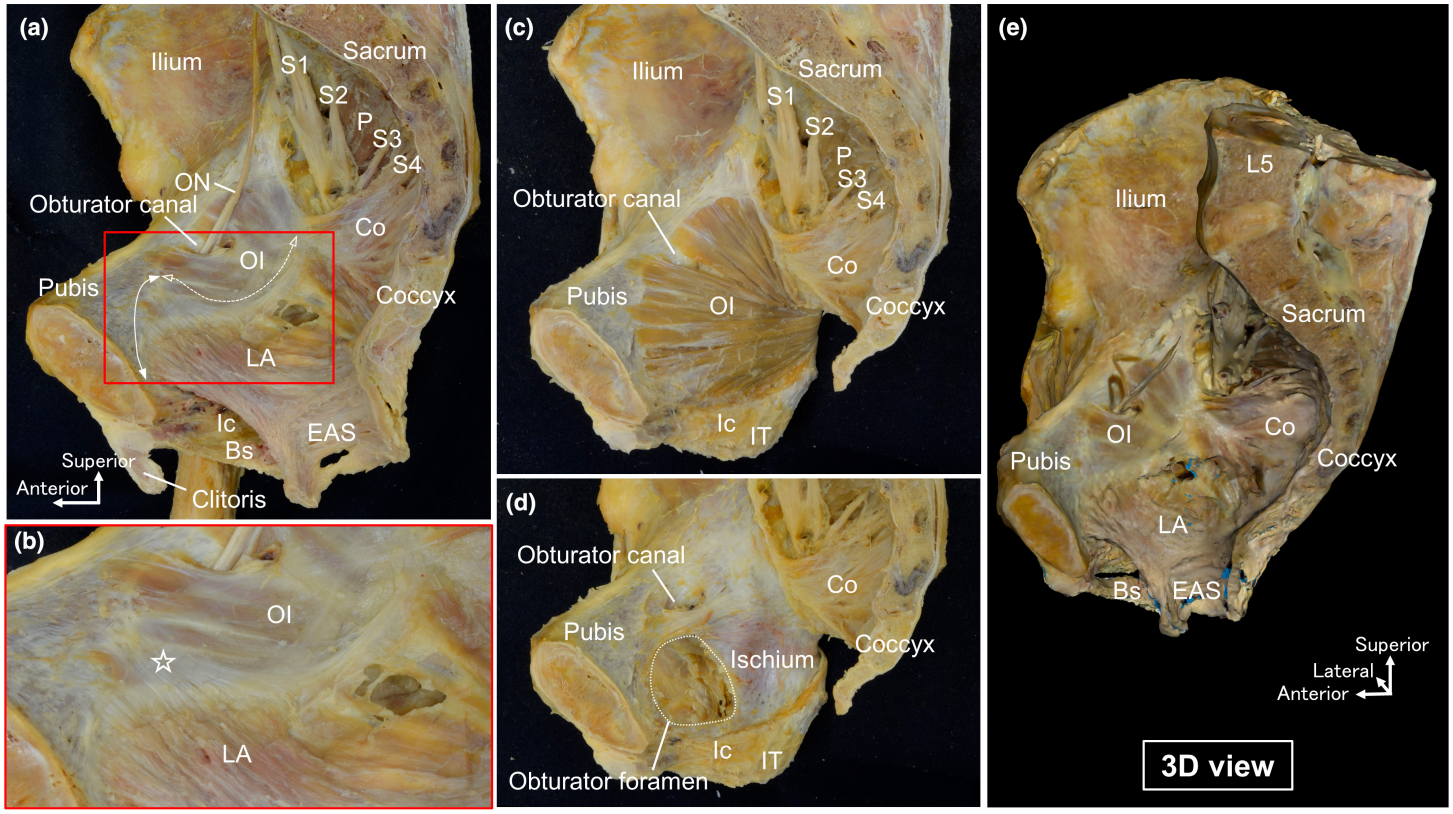
Relationship between the levator ani and obturator internus. (a) The female hemipelvis seen from the medial aspect after removal of the pelvic viscera to show the levator ani. The obturator internus is covered by the obturator fascia. Only the most superficial muscle bundle can be observed, with the anterior portion of the levator ani originating from the pubic bone (indicated by the double‐headed arrow line) and the posterior portion originating from the obturator fascia (indicated by the dotted line with empty arrowheads). (b) Magnified view of the red rectangular space in (a). The tendons of the levator ani are in contact with the obturator fascia (indicated by the star). (c) After removal of the levator ani and obturator fascia. The obturator internus consists of the pelvic wall. (d) After removal of the obturator internus and obturator membrane. The bone structures are also observed. (e) 3D scanned image (see Appendix). Bs, bulbospongiosus; Co, coccygeus; EAS, external anal sphincter; Ic, ischiocavernosus; IT, ischial tuberosity; LA, levator ani; OI, obturator internus; ON, obturator nerve; P, piriformis.

The levator ani formed the lateral and inferior walls of the pelvic cavity and was continuous with the coccygeus superiorly and the external anal sphincter inferiorly (Figure 1a, Appendix S1). The obturator internus, covered by the obturator fascia, was observed on the lateral side of the levator ani. Only the most superficial muscle bundle was observed from the medial aspect, with the anterior part of the levator ani originating from the pubic bone (indicated by the double‐headed arrow line in Figure 1a) and the posterior part originating from the obturator fascia (indicated by the dotted line with empty arrowheads in Figure 1a). In the region where the obturator internus overlapped the levator ani, the tendons of the levator ani were in contact with the obturator fascia (indicated by the star in Figure 1b). After removing the levator ani, the entire obturator internus was revealed (Figure 1c). The obturator internus, along with the coccygeus and piriformis, comprises the pelvic wall. After removing the obturator internus and obturator membrane, the pubis, ischium and obturator foramen were examined (Figure 1d).

**FIGURE 2:**
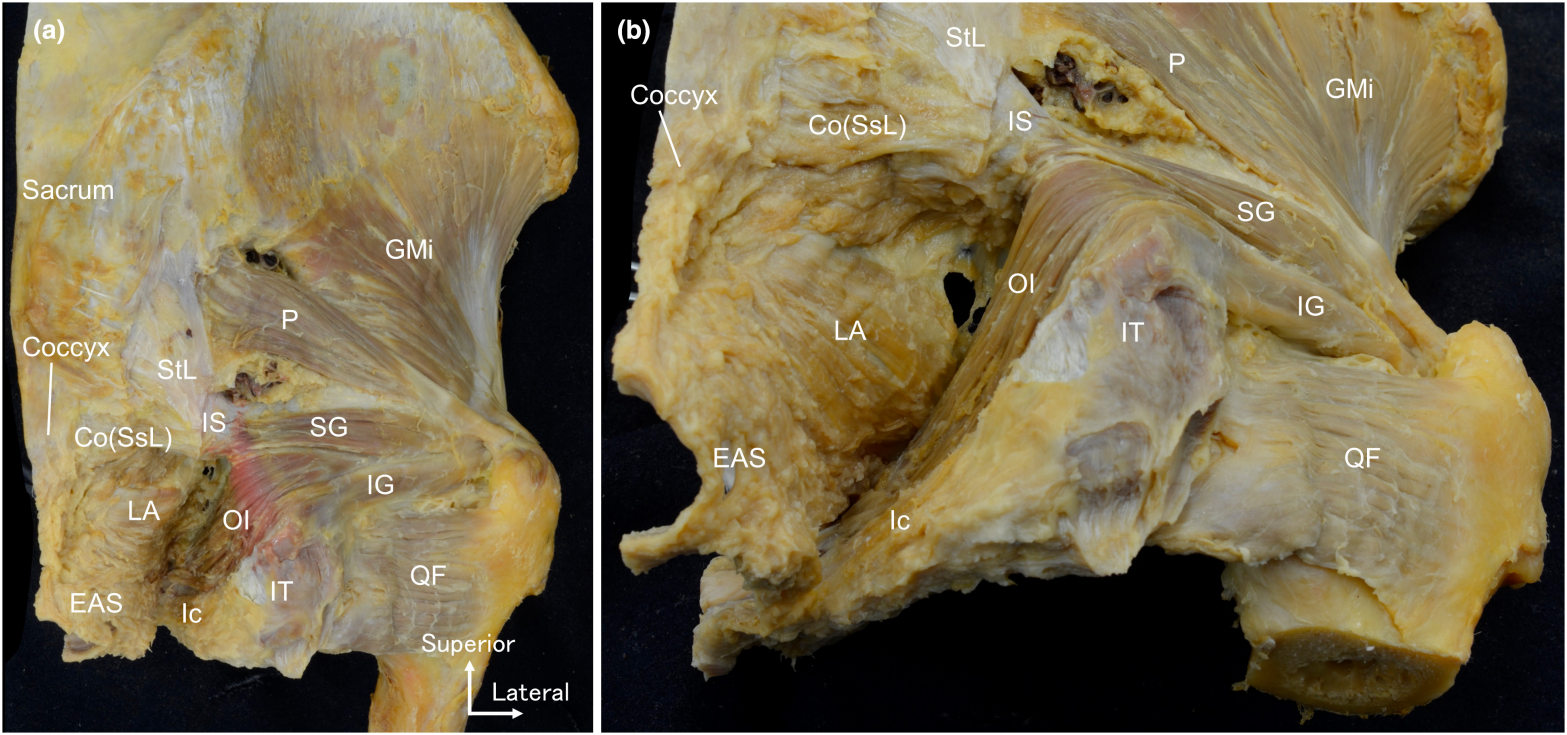
The levator ani and obturator internus located medial and lateral to the ischioanal fossa. (a) A female hemipelvis as seen from the posterior aspect. The rotator muscles of the hip joint and pelvic floor muscles were both observed. (b) Posteroinferior aspect. The obturator internus and levator ani are relative to each other through the ischioanal fossa. Co, coccygeus; EAS, external anal sphincter; GMi, gluteus minimus; Ic, ischiocavernosus; IG, inferior gemellus muscle; IS, ischial spine; IT, ischial tuberosity; LA, levator ani; OI, obturator internus; P, piriformis; QF, quadratus femoris muscle; SG, superior gemellus muscle; SsL, sacrospinous ligament; StL, sacrotuberous ligament.

In the posterior aspect, the ischioanal fossa was filled with loose connective tissue, mainly adipose tissue. Removal of the loose connective tissue and obturator fascia revealed the obturator internus and levator ani (Figure 2a). Additionally, the obturator internus and levator ani were located lateral and medial to the ischioanal fossa, respectively; that is, the obturator internus and levator ani were relative to each other through the ischioanal fossa (Figure 2b).

**FIGURE 3:**
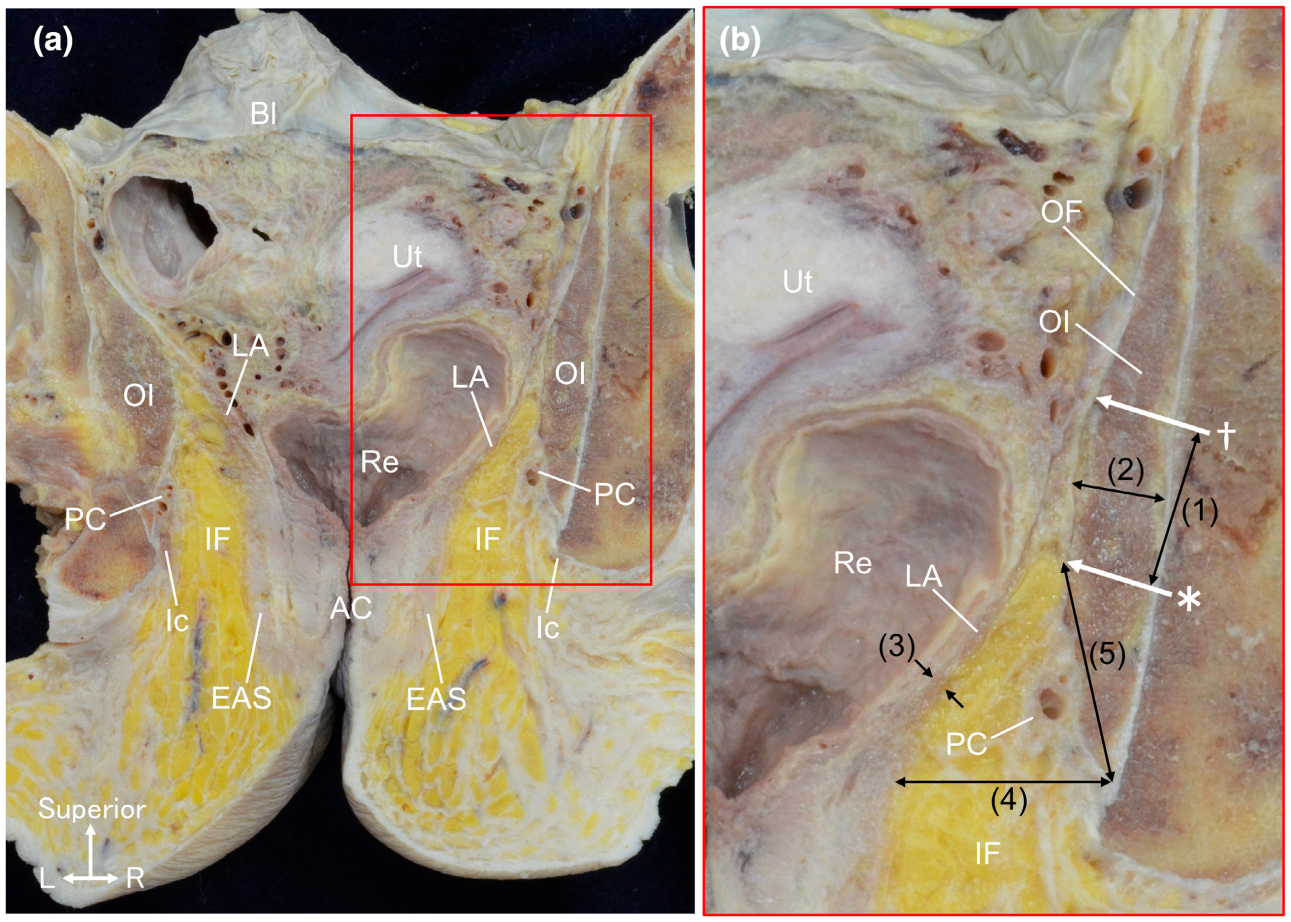
Wide contact distance between the obturator internus and levator ani. (a) The sectioned plane parallel to the anal canal in the female pelvis. Sections of the obturator internus and levator ani are observed. The ischioanal fossa is filled with loose connective tissue, mainly adipose tissue. (b) Magnified view of the red rectangular area in (a). The obturator fascia covers the medial surface of the obturator internus, and the levator ani is attached to the obturator fascia. Their contact area is wide superoinferiorly (the dagger indicates the superior end of the contact area, and the asterisk indicates the inferior end). AC, anal canal; Bl, bladder; EAS, external anal sphincter; Ic, ischiocavernosus; IF, ischioanal fossa; LA, levator ani; OF, obturator fascia; OI, obturator internus; PC, pudendal canal; Re, rectum; Ut, uterus.

In the coronal section through the anal canal, the ischioanal fossa was filled with loose connective tissue, mainly the adipose tissue, and the obturator internus and levator ani were located on the lateral and medial sides of the fossa, respectively (Figure 3a). The obturator fascia covered the medial surface of the obturator internus, and the levator ani was attached to the obturator fascia superior to the ischioanal fossa (Figure 3b). The contact area between the obturator internus and levator ani was wide superoinferiorly (the dagger indicates the superior end of the contact area, and the asterisk indicates the inferior end in Figure 3b). In this section, (1) the height of the contact area between the obturator internus and levator ani was 24.6 ± 9.1 mm, (2) the maximum thickness of the obturator internus (2) was 16.1 ± 5.3 mm, (3) the maximum thickness of the levator ani was 3.2 ± 1.0 mm, with the obturator internus being thicker, (4) the width of the ischioanal fossa was 19.3 ± 7.1 mm and (5) the depth of the ischioanal fossa was 25.4 ± 7.1 mm (Table 1).

**TABLE 1:** Measurement of the key structures in the plane parallel to the anal canal (cross‐section parallel to the muscle bundles of the levator ani)

	Mean [SD], mm
(1) Height of contact area between OI and LA	24.6 [9.1]
(1) Maximum thickness of OI	16.1 [5.3]
(2) Maximum thickness of LA	3.2 [1.0]
(3) Width of IF	19.3 [7.1]
(4) Depth of IF	25.4 [7.1]

Abbreviations: IF, ischioanal fossa; LA, levator ani; OI, obturator internus.

**FIGURE 4:**
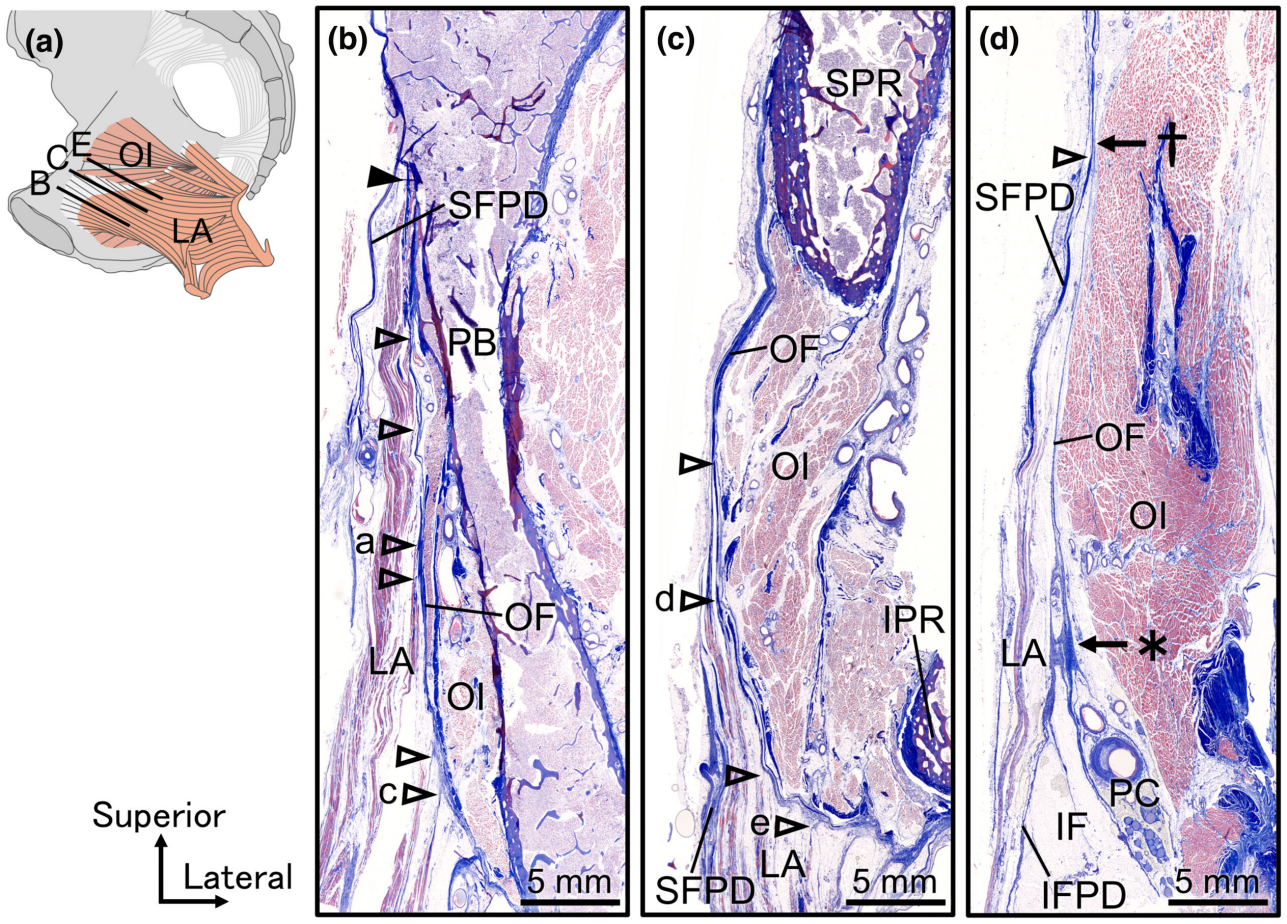
Visualisation of the obturator fascia and the origin of the levator ani using histological techniques. (a) Illustration of the female hemipelvis from the medial aspect. The black lines indicate the collection sites of the histological sections in (b, c, e). They were stained with Masson's trichrome. (b) Section at the level of the pubis body. The levator ani is attached to the bone of the body of the pubis in the superior portion (black arrowhead). However, in the inferior portion, several muscle layers of the levator ani are attached to the obturator fascia through the tendons (empty arrowheads). (c) Section at the level of the superior pubic ramus. All muscle fibres of the levator ani are attached to the obturator fascia via the tendons (indicated by the empty arrowheads). (d) Magnified view of the dotted rectangular space in (c). The origin of the levator ani is observed in the inferior region of the obturator fascia. (e) Section at the obturator canal level. The lateral surface of the levator ani is covered by the inferior fascia of the pelvic diaphragm and the obturator fascia. In this area, the obturator internus and levator ani share the obturator fascia. The dagger indicates the point where the superior fascia of the pelvic diaphragm joins the obturator fascia, and the asterisk indicates the point at which the inferior fascia of the pelvic diaphragm joins the obturator fascia. IF, ischioanal fossa; LA, levator ani; IFPD, inferior fascia of the pelvic diaphragm; IPR, inferior pubic ramus; OF, obturator fascia; OI, obturator internus; PB, pubic body (the body of the pubis); PC, pudendal canal; SFPD, superior fascia of the pelvic diaphragm; SPR, superior pubic ramus.

Cross‐sections parallel to the muscle fibres of the levator ani at the levels of the body of the pubis, superior pubic ramus and obturator canal were observed histologically (Figure 4a). In the section at the level of the body of the pubis, the obturator internus attached to the medial surface of the body of the pubis, and the levator ani was located further medial to it (Figure 4b). The medial surface of the obturator internus was covered by the obturator fascia, while the medial surface of the levator ani was covered by the superior fascia of the pelvic diaphragm. The levator ani was attached to the bone of the pubis in the superior portion (indicated by the black arrowhead in Figure 4b), whereas in the inferior portion, several muscle layers of the levator ani were attached to the obturator fascia through the tendons (indicated by the empty arrowheads in Figure 4b). In the section at the level of the superior pubic ramus, the obturator internus was located between the superior and inferior pubic rami, and the levator ani was located medial to it (Figure 4c). In this section, all muscle fibres of the levator ani were attached to the obturator fascia via the tendons (indicated by the empty arrowheads in Figure 4c). The origin of the levator ani was also observed in the inferior region of the obturator fascia in a position entirely different from the conventional ‘the tendinous arch of levator ani’ (indicated by the empty arrowhead with ‘e’ in Figure 4d). At the level of the obturator canal, the medial surface of the obturator internus was covered by the obturator fascia, the lateral surface of the levator ani was covered by the inferior fascia of the pelvic diaphragm, and the medial surface of the levator ani was covered by the superior fascia of the pelvic diaphragm (Figure 4d). The ischioanal fossa was surrounded laterally by the obturator fascia and medially by the inferior fascia of the pelvic diaphragm and was filled with adipose tissue. Superior to the pudendal canal, the inferior fascia of the pelvic diaphragm adjoined to the obturator fascia (indicated by the asterisk in Figure 4d), and 20–30 mm superior to it, the superior fascia of the pelvic diaphragm adjoined to the obturator fascia (indicated by the dagger in Figure 4d). Consequently, in the wide area between these confluence points, the lateral surface of the levator ani was covered by the obturator fascia and not by the inferior fascia of the pelvic diaphragm. In other words, the obturator internus and levator ani share the obturator fascia in this area. In addition, several muscle layers of the levator ani were broadly attached to this shared obturator fascia.

**FIGURE 5:**
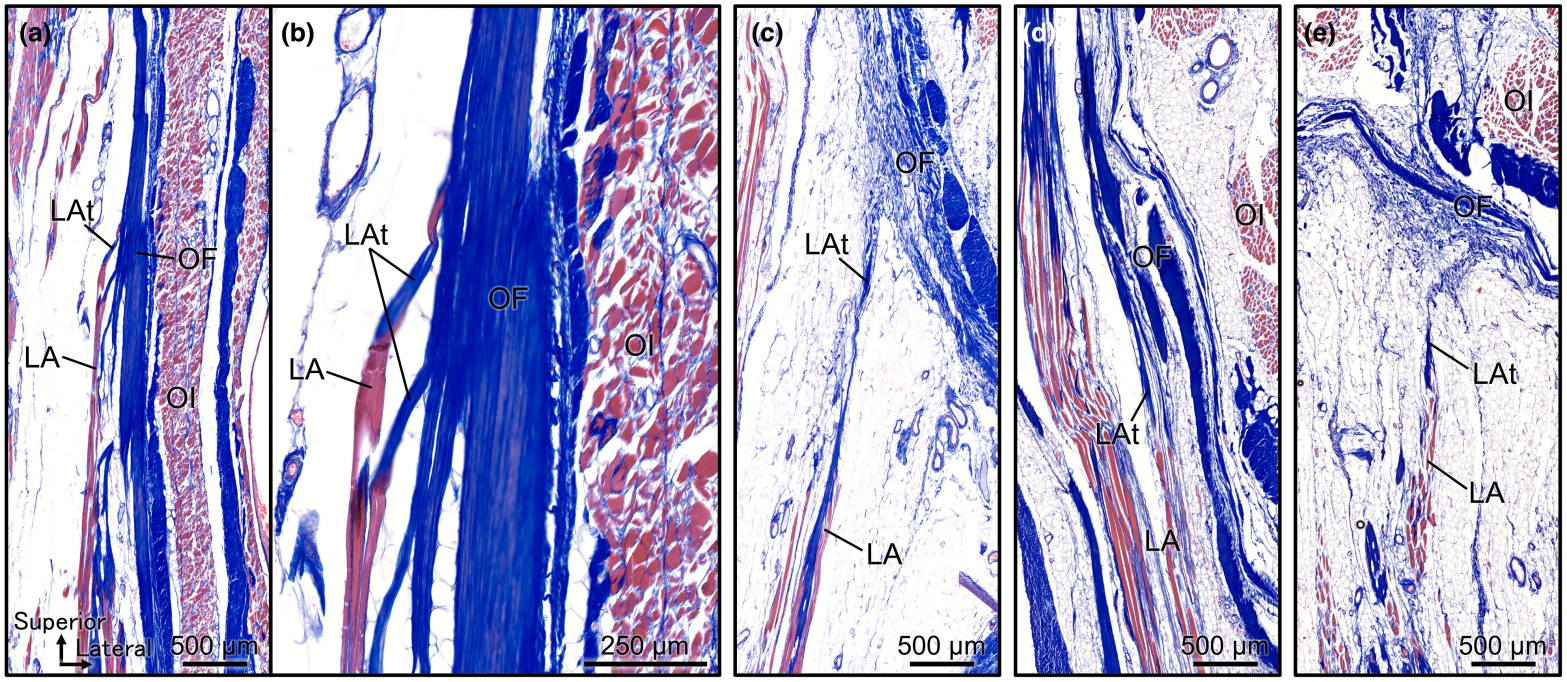
Attachments of the levator ani to the obturator fascia with collagen fibres merging. (a) Magnified image of the attachment point indicated by empty arrowhead ‘a’ in Figure 4b. The muscle fibres of LA migrated to tendons composed of collagen fibres, which were attached to OF (c–e shows similar findings). (b) A strongly magnified image of the attachment point of LAt to OF in (a). OF was formed as a distinct and thick layer of collagen fibres. The collagen fibres of LAt merged with the collagen fibres of OF. (c) Magnified image of the attachment point indicated by empty arrowhead ‘c’ in (b). (d) Magnified image of the attachment point indicated by empty arrowhead ‘d’ in (c). (e) Magnified image of the attachment point indicated by empty arrowhead ‘e’ in Figure 4c. LA, levator ani; LAt, tendon of levator ani; OF, obturator fascia; OI, obturator internus.

These attachment points of the levator ani to the obturator fascia are shown at high magnification in Figure 5a,c,d,e correspond to empty arrowheads a, c, d and e in Figure 4. The muscle fibres of the levator ani migrated into the tendons, composed of collagen fibres. However, the obturator fascia was also composed of collagen fibres and was formed as a distinct and thick layer of collagen fibres. The collagen fibres of the tendon of the levator ani and the collagen fibres of the obturator fascia merged and intermingled at the origin of the levator ani.

## 4 DISCUSSION

**FIGURE 6:**
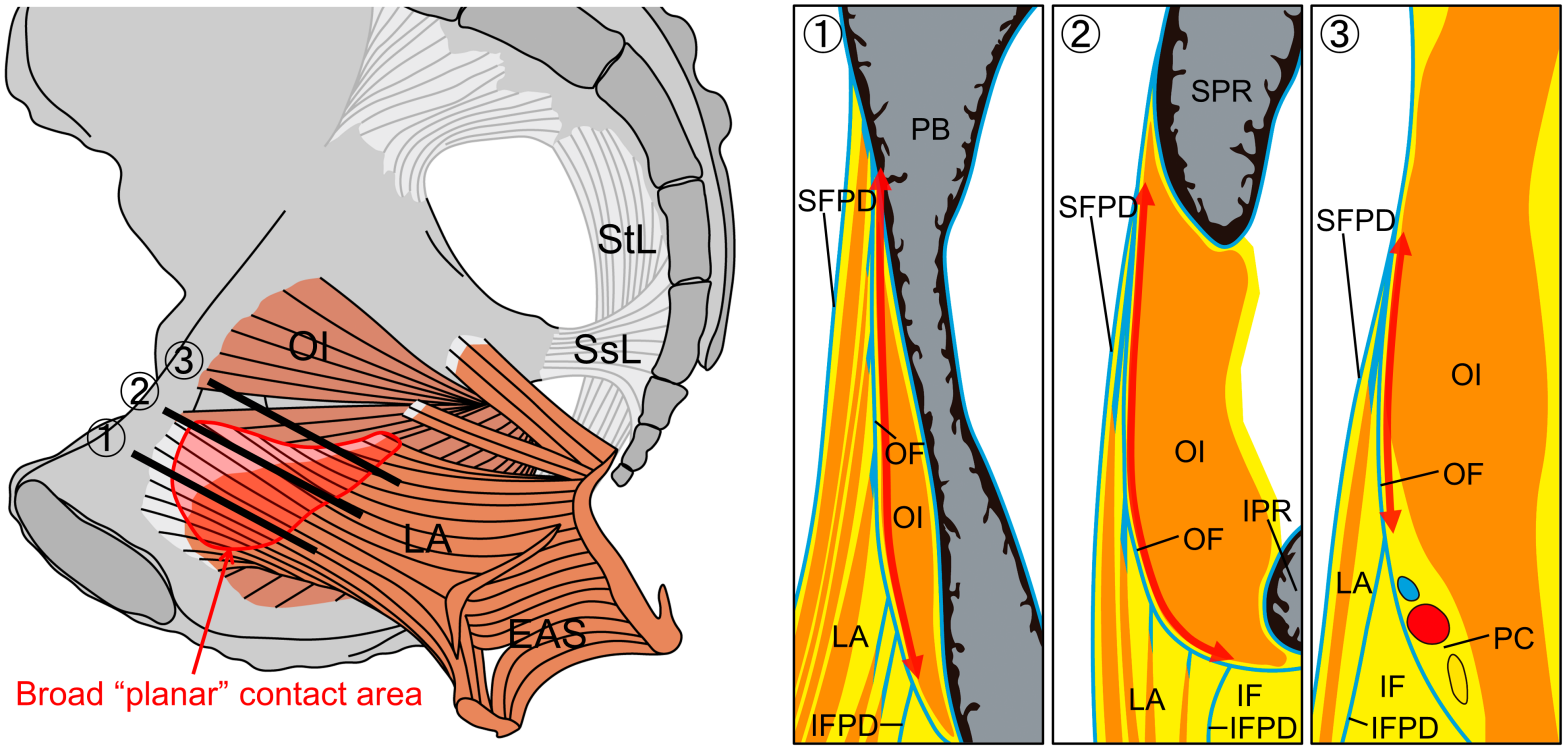
Broad ‘planar’ contact between the obturator internus and levator ani. The female hemipelvis is seen from the medial aspect, showing the obturator internus, which is one of the rotator muscles of the hip joint, and the levator ani, which is one of the pelvic floor muscles. The area in red represents the broad planar contact area between the obturator internus and levator ani. The three right sections are at the level of the (1) body of the pubis, (2) superior pubic ramus and (3) obturator canal and correspond to the three black lines in the left illustration. The obturator internus and levator ani share a large area of the obturator fascia, which serves as the attachment of several muscle layers of the levator ani. EAS, external anal sphincter; IF, ischioanal fossa; LA, levator ani; IFPD, inferior fascia of the pelvic diaphragm; IPR, inferior pubic ramus; OF, obturator fascia; OI, obturator internus; PB, pubic body (the body of pubis); PC, pudendal canal; SFPD, superior fascia of the pelvic diaphragm; SPR, superior pubic ramus; SsL, sacrospinous ligament; StL, sacrotuberous ligament.

This study clarified that the obturator internus and levator ani were in broad planar contact (Figure 6) and shared a large area of the obturator fascia, which provided the attachment of several muscle layers of the levator ani. Furthermore, inferior to the contact area between the obturator internus and levator ani, the ischioanal fossa extended, surrounded medially by the inferior fascia of the pelvic diaphragm and laterally by the obturator fascia and was filled with adipose tissue. Thus, the obturator internus and levator ani were interrelated, with broad contact through the obturator fascia superior to the ischioanal fossa.

Conventionally, it has been assumed that the anterior origin of the levator ani is the pubis and that the posterior origin is the thickened part of the obturator fascia (the tendinous arch of the levator ani) (Clemente, 1985; Cunningham & Romanes, 1981; Henle, 1866; PL, 1995; Standring & Gray, 2015; Tillaux, 1890). In other words, the point of contact between the levator ani and the obturator internus has been described as a linear attachment, localised to the posterior part of the levator ani. Although there have been reports on the attachment of the levator ani to the periosteum of the pubis by histological analysis (Kim et al., 2015), analysing the relationship between the levator ani and obturator internus is insufficient. This study revealed that the contact area between the levator ani and obturator internus runs anteroposteriorly and that they share a wide range of the obturator fascia with a superoinferior width of approximately 25 mm, touching as a ‘plane’. In addition, this planar contact area includes the attachments of several muscle layers of the levator ani to the obturator fascia. One of the ingenuities of this research method is the observation method of the cross‐section. Instead of the common coronal or transverse plane, the sectioning and observation were performed in the plane parallel to the anal canal, which is the cross‐section parallel to the muscle bundles of the levator ani. The direction of this cross‐section is consistent with the direction of muscle contraction. Such ingenuity in the sectioning plane enabled us to visualize the tendon and the relationship between its attachments and the fascia.

Several previous studies have suggested a functional and physiological relationship between the obturator internus and levator ani. Studies of randomized controlled trials on rehabilitation have reported that hip rehabilitation contributed to the strengthening of the pelvic floor muscles and the improvement of stress urinary incontinence (Jordre & Schweinle, 2014; Tuttle et al., 2016). Several studies, including a prospective cohort, reported that improved hip function with total hip arthroplasty improved urinary incontinence (Baba et al., 2014; Martines et al., 2022; Okumura et al., 2017; Tamaki et al., 2014). An electromyography study reported that electrophysiological stimulation of the fascia of the obturator internus resulted in the contraction of the levator ani (Chin et al., 2021). These studies suggest a functional and physiological link between the obturator internus and levator ani. To the best of our knowledge, the present study is the first to elucidate in detail the broad planar contact between these two muscles and to provide an anatomical basis for their linkage. Our findings suggest that the levator ani moves in conjunction with the obturator internus. The dynamic movement of the obturator internus with a thickness of approximately 16 mm probably acts on the levator ani with a thickness of approximately 3 mm through the obturator fascia, creating the foundation for the function of the levator ani and contributing to the support of the pelvic viscera. Therefore, an approach involving the hip joint muscles may be effective in improving defaecation/urinary function. In addition, the ischioanal fossa, a region between the obturator internus and levator ani that is approximately 20 mm thick and 25 mm deep, may change the structural positioning of these two muscles if the ischioanal fossa becomes fibrotic or degenerates, which may affect the movement function of the levator ani.

Recent anatomical studies have shown that the continuous sheet‐like structure of connective tissue around joints constitutes the intermuscular septum and provides an attachment to the surrounding muscles (tendons) and ligaments (Amaha et al., 2019; Hoshika et al., 2019; Nasu et al., 2020; Tsutsumi et al., 2020). In this way, connective tissue can form a continuous structure with a three‐dimensional expanse, providing a ‘field’ for multiple muscles and ligaments to exert their functions. Furthermore, this study suggests that the connective tissue, such as the obturator fascia, also plays a vital role between the obturator internus and levator ani. The fascia, such as the fibrous membranous structure, probably mediates the movement of the obturator internus and levator ani, creating a field for the levator ani muscle to exert its function.

The levator ani is important for supporting the pelvic organs and defaecation/urinary functions (Standring & Gray, 2015). It is the largest of the pelvic floor muscles and has various muscle bundles. The anterior part of the levator ani has been suggested to be structurally and functionally significant. Since the anterior part of the levator ani is mainly inserted into the visceral wall, some researchers have named this area the ‘pubovisceralis’ and emphasised it as the main part of the pelvic floor muscle (Kearney et al., 2004; Kim et al., 2015). In addition, other anatomical studies have shown that the muscle bundles of the anterior portion form slings in front of and behind the anal canal and that its muscle fibres are continuous with the urethral sphincter (Baramee et al., 2020; Muro et al., 2021; Suriyut et al., 2020). These anatomical findings suggest that the anterior part of the levator ani is involved in defaecation/urinary function. Previously, the anterior part of the levator ani was considered to be attached only to the pubis (Cunningham & Romanes, 1981; Kearney et al., 2004; Kim et al., 2015; Standring & Gray, 2015). However, the present study clarified that the anterior part of the levator ani was attached not only to the pubis but also to the obturator fascia, with the muscle origin of several muscle layers through the tendons. Therefore, when considering the function of the anterior part of the levator ani, it is necessary to consider not only its attachment to the pubis but also its attachment to the obturator fascia. Therefore, the anterior part of the levator ani is probably affected by the obturator internus as well as the posterior portion.

One of the approaches to pelvic floor function based on hip joint movement is positioning to improve constipation. The recommended position for improving constipation defaecation is the squatting position (‘The Thinker’ position) (Modi et al., 2019; Sakakibara et al., 2010; Takano & Sands, 2016). The improvement in constipation by squatting has conventionally been explained only by the relaxation of the puborectalis (part of the levator ani) associated with hip joint flexion. However, the anatomical relationship between hip motion and relaxation of the levator ani remains unclear. The present study clarified the structural relationship between the obturator internus, a hip joint muscle and the levator ani. The squatting position seems to cause not only hip flexion but also abduction and external rotation, which may affect the levator ani through contraction of the obturator internus. Focusing on the obturator internus may lead to new analyses and developments regarding defaecation positioning.

Our study has a few limitations. First, the ages of the subjects were skewed because the cadavers used in this study were elderly adults with an average age of >70. Hence, the muscle fibres may be more atrophied than those in younger cadavers. Second, this study was purely anatomical; therefore, we were unable to provide quantitative measurements of the movement of the obturator internus and levator ani. In the future, biomechanical studies may provide additional information on the functional linkage between these two muscles.

## 5 CONCLUSION

The obturator internus and levator ani share a broad planar contact through the obturator fascia. These anatomical findings suggest that the movement of the obturator internus probably acts on the levator ani through the obturator fascia, creating the foundation for the function of the levator ani and contributing to the support of the pelvic viscera. This may provide an anatomical basis for the effectiveness of the hip muscles in improving defaecation/urinary function through enabling balanced and proper movements.

## AUTHOR CONTRIBUTIONS

SM contributed to the conception and design of the work; acquisition, analysis and interpretation of data; drafting of the manuscript and final approval of the version to be published. AM and TI contributed to the analysis and interpretation of data, critical revision of the draft and provided final approval of the version to be published. KC, MN and KA contributed to the interpretation of data, critical revision of the draft and provided final approval of the version to be published.

## FUNDING INFORMATION

This work was supported by JSPS KAKENHI (grant nos. JP19K23821, JP21K15329 and JP21H03799).

## CONFLICT OF INTEREST

The authors declare no financial disclosure and conflict of interest.

## INSTITUTIONAL REVIEW BOARD APPROVAL

The Board of Ethics at Tokyo Medical and Dental University approved the study (approval number: M2018‐006). All methods were performed following the relevant guidelines and regulations.

## Supporting information


Appendix S1.

## Data Availability

Data supporting this study's findings are available from the corresponding author on a reasonable request.
