# Supplementary material for: Anatomical basis for contribution of hip joint motion by the obturator internus to defaecation/urinary functions by the levator ani via the obturator fascia
Source: J Anat. 2022 Dec 18;242(4):657–65. doi: 10.1111/joa.13810 (PMC10008353; doi:10.1111/joa.13810)
Supplement: Supplementary file 1 — Appendix S1. [file JOA-242-657-s001.pdf]

この領域を表示するためには、Adobe Readerなどの3D PDF対応ビューワーが必要です。
